# Supplementary material for: Efficacy of Exercise on Muscle Function and Physical Performance in Older Adults with Sarcopenia: An Updated Systematic Review and Meta-Analysis
Source: Int J Environ Res Public Health. 2022 Jul 5;19(13):8212. doi: 10.3390/ijerph19138212 (PMC9266336; doi:10.3390/ijerph19138212)
Supplement: Supplementary file 1 [file ijerph-19-08212-s001.zip › Supplementary S1.pdf]

Supplementary S1

Search strategy

PubMed (abstract/title/keywords)

(("sarcopenia"[Title/Abstract] OR "sarcopen*"[Title/Abstract]) AND ("exercise"[Title/Abstract] OR "exercis*"[Title/Abstract] OR ("exercis*"[Title/Abstract] AND ("endurance"[Title/Abstract] OR "aerobic"[Title/Abstract] OR "resistance"[Title/Abstract] OR "balance"[Title/Abstract] OR "flexibility"[Title/Abstract] OR "combi*"[Title/Abstract] OR "mixed"[Title/Abstract] OR "multi*"[Title/Abstract] OR "eccentric"[Title/Abstract] OR "eccentric"[Title/Abstract] OR "concentric"[Title/Abstract] OR "isometric"[Title/Abstract] OR ("plyometric"[All Fields] OR "plyometrics"[All Fields]))) OR "walk*"[Title/Abstract] OR "run"[Title/Abstract]) AND ("muscle mass"[Title/Abstract] OR "muscle strength"[Title/Abstract] (("sarcopenia"[Title/Abstract] OR "sarcopen*"[Title/Abstract]) AND ("exercise"[Title/Abstract] OR "exercis*"[Title/Abstract] OR ("exercis*"[Title/Abstract] AND ("endurance"[Title/Abstract] OR "aerobic"[Title/Abstract] OR "resistance"[Title/Abstract] OR "balance"[Title/Abstract] OR "flexibility"[Title/Abstract] OR "combi*"[Title/Abstract] OR "mixed"[Title/Abstract] OR "multi*"[Title/Abstract] OR "eccentric"[Title/Abstract] OR "eccentric"[Title/Abstract] OR "concentric"[Title/Abstract] OR "isometric"[Title/Abstract] OR ("plyometric"[All Fields] OR "plyometrics"[All Fields]))) OR "walk*"[Title/Abstract] OR "run"[Title/Abstract]) AND ("muscle mass"[Title/Abstract] OR "muscle strength"[Title/Abstract] OR "muscle function"[Title/Abstract] OR "fat-free mass"[Title/Abstract] OR "lean mass"[Title/Abstract] OR ("musc*"[Title/Abstract] AND ("function*"[Title/Abstract] OR "power"[Title/Abstract] OR "size"[Title/Abstract] OR "volume"[Title/Abstract] OR "thick*"[Title/Abstract] OR "enlarge*"[Title/Abstract] OR ("frailty"[MeSH Terms] OR "frailty"[All Fields] OR "weakness"[All Fields] OR "weaknesses"[All Fields]))) OR ("physical"[Title/Abstract] AND ("fitness"[Title/Abstract] OR "function*"[Title/Abstract] OR "capacity"[Title/Abstract] OR "disability"[Title/Abstract] OR ("perform"[All Fields] OR "performable"[All Fields] OR "performance"[All Fields] OR "performance s"[All Fields] OR "performances"[All Fields] OR "performative"[All Fields] OR "performatively"[All Fields] OR "performatives"[All Fields] OR "performativities"[All Fields]

OR "performativity"[All Fields] OR "performed"[All Fields] OR "performer"[All Fields] OR "performer s"[All Fields] OR "performers"[All Fields] OR "performing"[All Fields] OR "performs"[All Fields])))) AND ("aged"[Title/Abstract] OR "aging"[Title/Abstract] OR "geriatric*"[Title/Abstract] OR "old"[Title/Abstract] OR "ageing"[Title/Abstract] OR "senior"[Title/Abstract] OR "elder*"[Title/Abstract] OR "adult*"[Title/Abstract])) AND ((fha[Filter]) AND (clinicaltrial[Filter]) AND (humans[Filter]) AND (english[Filter])) OR "muscle function"[Title/Abstract] OR "fat-free mass"[Title/Abstract] OR "lean mass"[Title/Abstract] OR ("musc*"[Title/Abstract] AND ("function*"[Title/Abstract] OR "power"[Title/Abstract] OR "size"[Title/Abstract] OR "volume"[Title/Abstract] OR "thick*"[Title/Abstract] OR "enlarge*"[Title/Abstract] OR ("frailty"[MeSH Terms] OR "frailty"[All Fields] OR "weakness"[All Fields] OR "weaknesses"[All Fields]))) OR ("physical"[Title/Abstract] AND ("fitness"[Title/Abstract] OR "function*"[Title/Abstract] OR "capacity"[Title/Abstract] OR "disability"[Title/Abstract] OR ("perform"[All Fields] OR "performable"[All Fields] OR "performance"[All Fields] OR "performance s"[All Fields] OR "performances"[All Fields] OR "performative"[All Fields] OR "performatively"[All Fields] OR "performatives"[All Fields] OR "performativities"[All Fields] OR "performativity"[All Fields] OR "performed"[All Fields] OR "performer"[All Fields] OR "performer s"[All Fields] OR "performers"[All Fields] OR "performing"[All Fields] OR "performs"[All Fields])))) AND ("aged"[Title/Abstract] OR "aging"[Title/Abstract] OR "geriatric*"[Title/Abstract] OR "old"[Title/Abstract] OR "ageing"[Title/Abstract] OR "senior"[Title/Abstract] OR "elder*"[Title/Abstract] OR "adult*"[Title/Abstract])) AND ((fha[Filter]) AND (clinicaltrial[Filter]) AND (humans[Filter]) AND (english[Filter]))
(N = 145)

Web of Science (Topic)

S1: sarcopenia OR sarcopen*

S2: exercise OR exercis* OR (exercis* AND (endurance OR aerobic OR resistance OR balance OR flexibility OR combi* OR mixed OR multi* OR eccentric OR eccentric OR concentric OR isometric OR plyometric)) OR walk* OR run*

S3: “muscle mass” OR “muscle strength” OR “muscle function” OR “fat-free mass” OR “lean mass” OR (musc* AND (function* OR power OR size OR volume OR thick* OR enlarge* OR weakness)) OR (physical AND (fitness OR function* OR capacity OR disability OR performance))

S4: aged OR aging OR geriatric* OR old* OR ageing OR senior OR elder* OR adult*

S5: S1+S2+S3+S4 (N = 1976)

S6: Filters– English; article;

NOT Title=(relation* OR correlation OR study protocol OR healthy OR cross* OR associat* OR longitudinal OR mouse OR rat OR mice OR update)

S7: S5 + S6 (N = 1020)

EBSCOhost (Text All) (includes Medline, PsycINFO and sportdiscu)

S1: sarcopenia OR sarcopen*

S2: exercise OR exercis* OR (exercis* AND (endurance OR aerobic OR resistance OR balance OR flexibility OR combi* OR mixed OR multi* OR eccentric OR eccentric OR concentric OR isometric OR plyometric)) OR walk* OR run*

S3: “muscle mass” OR “muscle strength” OR “muscle function” OR “fat-free mass” OR “lean mass” OR (musc* AND (function* OR power OR size OR volume OR thick* OR enlarge* OR weakness)) OR (physical AND (fitness OR function* OR capacity OR disability OR performance))

S4: aged OR aging OR geriatric* OR old* OR ageing OR senior OR elder* OR adult*

S5: S1+S2+S3+S4

S6: Filters– English; academic journals; aged 65+ years

S7: NOT Title=(relation* OR correlation OR study protocol OR healthy OR cross* OR associat* OR longitudinal OR mouse OR rat OR mice OR update)

S8: S6 + S7 (N = 1175)

ProQuest

noft(sarcopenia OR sarcopen*) AND noft(exercise OR exercis* OR (exercis* AND (endurance OR aerobic OR resistance OR balance OR flexibility OR combi* OR mixed OR multi* OR eccentric OR eccentric OR concentric OR isometric OR plyometric)) OR walk* OR run*) AND noft("muscle mass" OR "muscle strength" OR "muscle function" OR "fat-free mass" OR "lean mass" OR (musc* AND (function* OR power OR size OR volume OR thick* OR enlarge* OR weakness)) OR (physical AND (fitness OR function* OR capacity OR disability OR performance))) AND noft(aged OR aging OR geriatric* OR old* OR ageing OR senior OR elder* OR adult*) NOT ti((relation* OR correlation OR study protocol OR healthy OR cross* OR associat* OR longitudinal OR mouse OR rat OR mice OR update))

(N = 869)

Scopus

(TITLE-ABS-KEY (sarcopenia OR sarcopen*) AND TITLE-ABS-KEY (exercise OR exerc is* OR (exercis* AND (endurance OR aerobic OR resistance OR balance OR flexibility OR combi* OR mixed OR multi* OR eccentric OR eccentric OR concentric OR isometric OR plyometric)) OR walk* OR run*) AND TITLE-ABS-KEY (“muscle AND mass” O R “muscle AND strength” OR “muscle AND function” OR “fatfree AND mass” OR “lean AND mass” OR (musc* AND (function* OR power OR size OR volume OR thick* O R enlarge* OR weakness)) OR (physical AND (fitness OR function* OR capacity OR d isability OR performance))) AND TITLE-ABS-KEY (aged OR aging OR geriatric* OR o ld* OR ageing OR senior OR elder* OR adult*) AND NOT TITLE ((relation* OR correlation OR study AND protocol OR healthy OR cross* OR associat* OR longitudinal OR mouse OR rat OR mice OR update)))

(N = 776)

Cochrane library

S1: sarcopenia OR sarcopen*

S2: exercise OR exercis* OR (exercis* AND (endurance OR aerobic OR resistance OR balance OR flexibility OR combi* OR mixed OR multi* OR eccentric OR eccentric OR concentric OR isometric OR plyometric)) OR walk* OR run*

S3: “muscle mass” OR “muscle strength” OR “muscle function” OR “fat-free mass” OR “lean mass” OR (musc* AND (function* OR power OR size OR volume OR thick* OR enlarge* OR weakness)) OR (physical AND (fitness OR function* OR capacity OR disability OR performance))

S4: aged OR aging OR geriatric* OR old* OR ageing OR senior OR elder* OR adult*

S5: S1+S2+S3+S4

S6: Filters–trials

S7: S5 + S6 (N = 846)

EMBASE

(sarcopenia:ab,ti OR sarcopen*:ab,ti) AND (exercise:ab,ti OR exercis*:ab,ti OR (exercis*:ab,ti AND (endurance:ab,ti OR aerobic:ab,ti OR resistance:ab,ti OR balance:ab,ti OR flexibility:ab,ti OR combi*:ab,ti OR mixed:ab,ti OR multi*:ab,ti OR eccentric:ab,ti OR concentric:ab,ti OR isometric:ab,ti OR plyometric:ab,ti)) OR walk*:ab,ti OR run*:ab,ti) AND ('muscle mass':ab,ti OR 'muscle strength':ab,ti OR 'muscle function':ab,ti OR 'fat-free mass':ab,ti OR 'lean mass':ab,ti OR (musc*:ab,ti AND (function*:ab,ti OR power:ab,ti OR size:ab,ti OR volume:ab,ti OR thick*:ab,ti OR enlarge*:ab,ti OR weakness:ab,ti)) OR (physical:ab,ti AND (fitness:ab,ti OR function*:ab,ti OR capacity:ab,ti OR disability:ab,ti OR performance:ab,ti))) AND (aged:ab,ti OR aging:ab,ti OR geriatric*:ab,ti OR old*:ab,ti OR ageing:ab,ti OR senior:ab,ti OR elder*:ab,ti OR adult*:ab,ti) NOT (relation*:ti OR correlation:ti OR 'study protocol':ti OR healthy:ti OR cross*:ti OR associat*:ti OR longitudinal:ti OR mouse:ti OR rat:ti OR mice:ti OR update:ti)

Filters– English; article；

(N = 533)

维普同义词扩展：骨骼肌减少症+sarcopenia+肌肉减少症+少肌症+肌少症

((((((((题名或关键词=骨骼肌减少症 OR 题名或关键词=sarcopenia) OR 题名或关键词=肌肉减少症) OR 题名或关键词=少肌症) OR 题名或关键词=肌少症) AND 题名或关键词=运动) AND (题名或关键词=老人 OR 题名或关键词=老年人)) AND (((文摘=RCT OR 文摘=随机对照) OR 文摘=随机分配) OR 文摘=随机))

（N=10）

知网

（（（主题=肌少症 或者 题名=肌少症 或者 v_subject=中英文扩展(肌少症) 或者 title=中英文扩展(肌少症)) 或者 (主题=肌肉减少症 或者 题名=肌肉减少症 或者 v_subject=中英文扩展(肌肉减少症) 或者 title=中英文扩展(肌肉减少症))） 或者 (主题=骨骼肌减少症 或者 题名=骨骼肌减少症 或者 v_subject=中英文扩展(骨骼肌减少症) 或者 title=中英文扩展(骨骼肌减少症))） 并且 （（（（主题=运动 或者 题名=运动 或者 v_subject=中英文扩展(运动) 或者 title=中英文扩展(运动)) 或者 (主题=有氧运动 或者 题名=有氧运动 或者 v_subject=中英文扩展(有氧运动) 或者 title=中英文扩展(有氧运动))） 或者 (主题=锻炼 或者 题名=锻炼 或者 v_subject=中英文扩展(锻炼) 或者 title=中英文扩展(锻炼))） 或者 (主题=阻力运动 或者 题名=阻力运动 或者 v_subject=中英文扩展(阻力运动) 或者 title=中英文扩展(阻力运动))） 并且 （（主题=老年人 或者 题名=老年人 或者 v_subject=中英文扩展(老年人) 或者 title=中英文扩展(老年人)) 或者 (主题=老人 或者 题名=老人 或者 v_subject=中英文扩展(老人) 或者 title=中英文扩展(老人))） 并且 （（（摘要=随机对照试验 或者 abstract_en=中英文扩展(随机对照试验)) 或者 (摘要=随机对照 或者 abstract_en=中英文扩展(随机对照))） 或者 (摘要=中英文扩展(RCT) 或者 abstract_en=RCT)）(模糊匹配)

（N=5）
