# Supplementary material for: Efficacy of Exercise on Muscle Function and Physical Performance in Older Adults with Sarcopenia: An Updated Systematic Review and Meta-Analysis
Source: Int J Environ Res Public Health. 2022 Jul 5;19(13):8212. doi: 10.3390/ijerph19138212 (PMC9266336; doi:10.3390/ijerph19138212)
Supplement: Supplementary file 1 [file ijerph-19-08212-s001.zip › Supplementary S2.pdf]

Supplementary S2

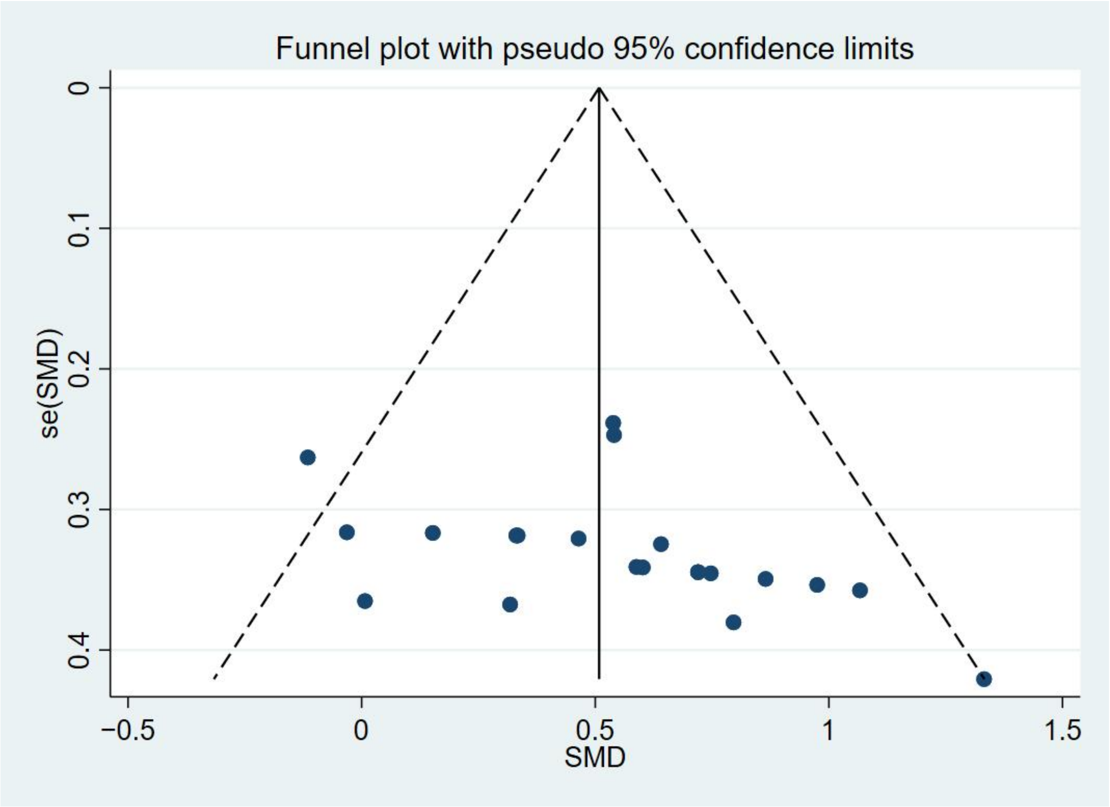

Figure S1. Funnel plot of knee extension strength

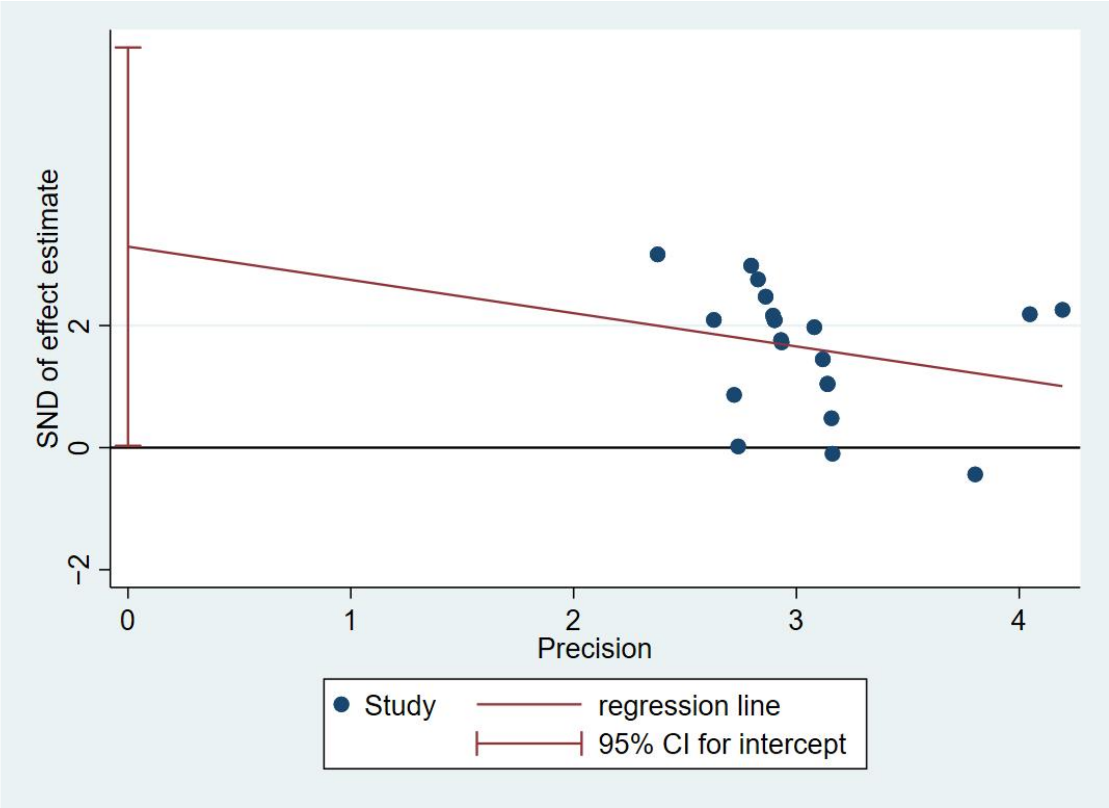

Figure S2. Egger plot of knee extension strength
